# Seaweeds and Their Secondary Metabolites: A Promising Drug Candidate With Novel Mechanisms Against Cancers and Tumor Angiogenesis

**DOI:** 10.7759/cureus.66662

**Published:** 2024-08-12

**Authors:** Taniya Mary Martin, Meenakshi Sundaram K

**Affiliations:** 1 Zebrafish Facility, Department of Anatomy, Saveetha Dental College and Hospitals, Saveetha Institute of Medical and Technical Sciences (SIMATS) Saveetha University, Chennai, IND

**Keywords:** seaweed, epigenetics, cytotoxicity, polyphenols, secondary metabolites, anticancer

## Abstract

Cancer continually remains a severe threat to public health and requires constant demand for novel therapeutic drug candidates. Due to their multi-target orientation, lesser toxicity, and easy availability, natural compounds attract more attention from current scientific research interest than synthetic drug molecules. The plants and microorganisms produce a huge variety of secondary metabolites because of their physiological diversification, and the seaweeds occupy a prominent position as effective drug resources. Seaweeds comprise microscopic or macroscopic photosynthetic, multicellular, eukaryotic marine algae that commonly inhabit the coastal regions. Several molecules (such as polysaccharides, lipids, proteinaceous fractions, phenolic compounds, and alkaloids) are derived from seaweeds, and those small molecules are well attractive and more effective in cancer research programs. Their structural variation, derivative diversity, and quantity vary with seaweed species and geographical origin. Their smaller molecular weight, unique derivatives, hydrophobicity, and degree of sulfation are reported to be causes of their crucial role against different cancer cells in vitro. Several reports showed that those compounds selectively discriminate between normal and cancer cells based on receptor variations, enzyme deficiency, and structural properties. The present review aimed to give a concise explanation regarding their structural diversity, extractability, and mechanism of action related to their anti-cancer activities based on recently published data.

## Introduction and background

**Figure 1:**
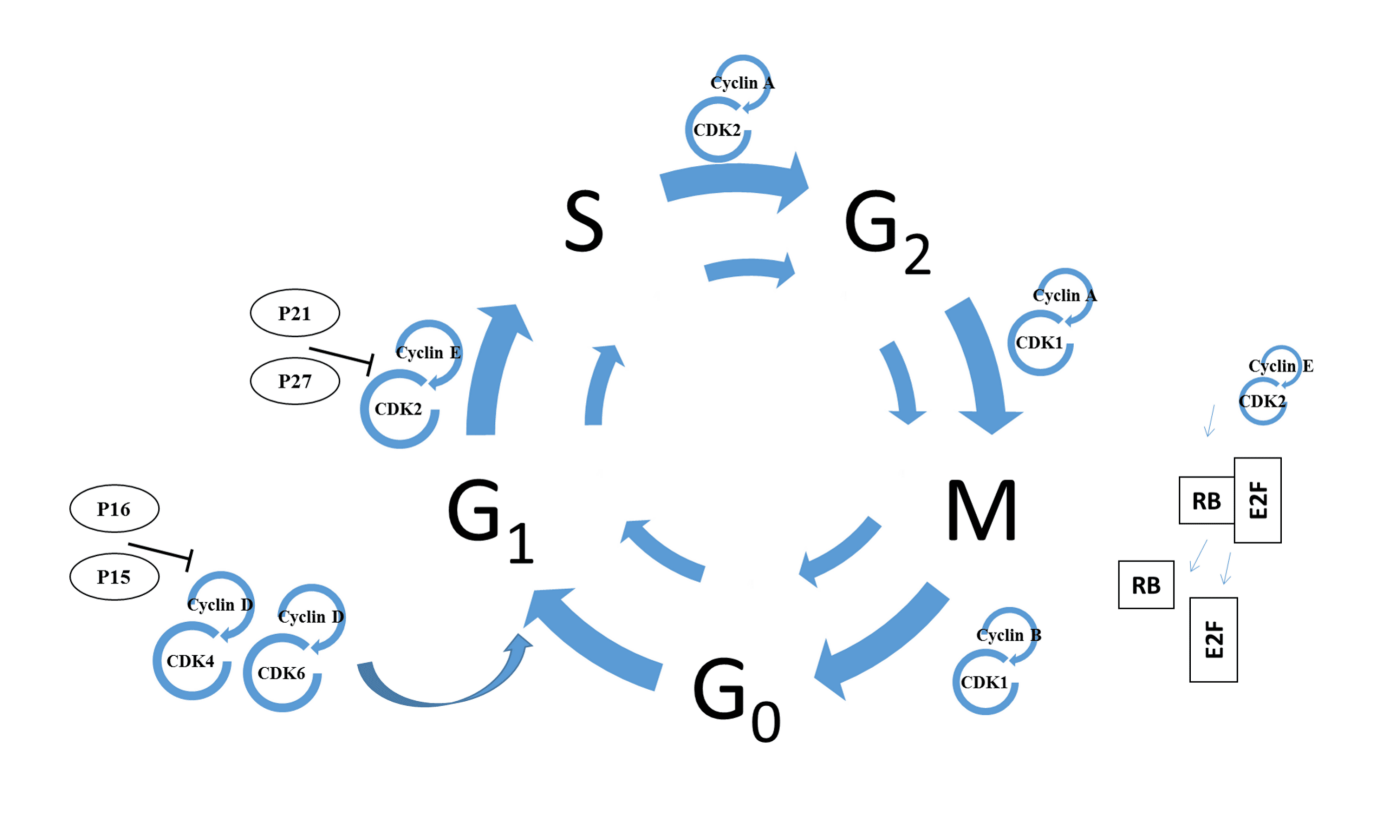
Cell cycle regulation and checkpoints The cell cycle is a highly regulated process that results in two daughter cells from a single one and consists of four stages, namely, gap stage (G1), DNA (deoxyribonucleic acid) synthesis (S), gap phase II (G2), and mitosis stage (M), respectively. Those stages are controlled and regulated by the heterodimeric coordination of cyclins (-A, -B, -C, -E) and cyclin-dependent kinases (CDK -1, -2, -4, -6, -8, -12). Many checkpoints (G1/S, G2/M, G1, and S) exist in the cell cycle to ensure smooth progression. p21 and p27 are prominent regulators in the G1 and S stages. Usually, any defect beyond the DNA repair at the S stage will induce those regulators and activate the programmed cell death pathways (apoptosis). The tumors evolved to break the normal regulators and apoptosis-influencing factors. Those checkpoints and regulators serve as drug targets for anticancer research. Image credit: Meenakshi Sundaram K.

**Figure 2:**
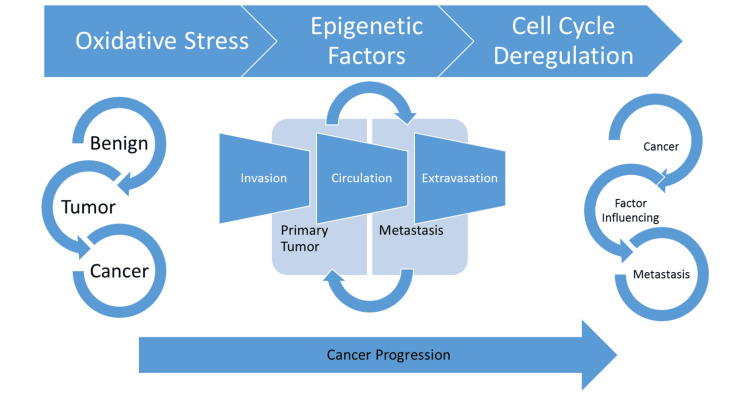
Summary of cancer progression Lifestyle modifications and other environmental factors strongly influence many metabolic imbalances, and these alterations further severely affect the equilibrium of the inflammation and oxidative stress conditions. Collectively, those complications alter the benign cells into tumor cells (cells with unregulated and disrupted cell proliferative activity). Tumors derive the ability to bypass normal cell signaling and become cancerous. Hence, it leads to the development of unconditional blood vessel formation (cancer angiogenesis) (Rb: retinoblastoma, p21, and p27: cyclin-dependent kinase inhibitors). Image credits: Meenakshi Sundaram K.

Cancer remains a huge challenge besides the constant development of its curatives and preventive measures due to its recurrence and metastatic behavior. The cancer cells only differ from other body cells by having improper signaling and inadequate checkpoints. These potentiate the benign tumors to form malignancy, resulting in metastasis (Figure 1). Because of physiological similarities, cancer cells further provide complications such as indistinguishable pathways and related molecular mechanisms that require fine-tuning before undergoing any treatment options (Figure 2). The current therapeutic options contain chemotherapy, radiotherapy, and a number of chemically derived drugs, but most of them put the patients at their maximum risk by means of side effects. Hence, the current scientific research firmly focuses on alternative therapeutic options against cancer metabolism. On comparing the synthetic drugs, the bioactive molecules have higher specificity on drug targets due to their well-defined three-dimensional structures and with fewer or lesser adverse side effects. The naturally derived molecules have been used since time immemorial to the present, and nearly 80% of people from developing or underdeveloped countries rely only on those compounds for their medicinal applications. The oceans fulfill the dietary requirements of three-fourths of the world's population, and marine organisms are considered to also be a more important source of biologically active products across the world [1-5].

Chemical diversity of the seaweed compounds

**Figure 3:**
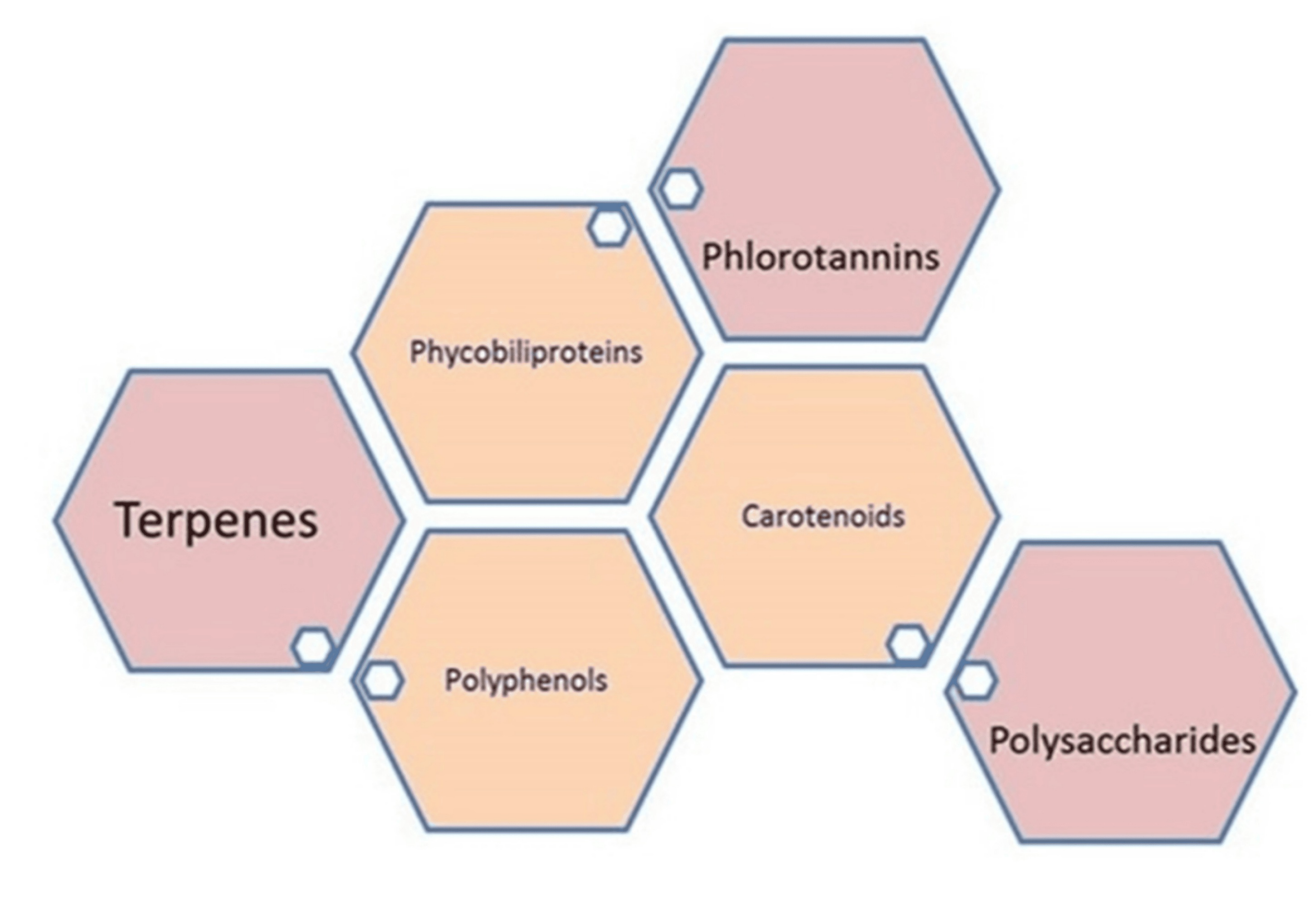
Seaweeds contain rich chemical diversity in view of pharmacological activities. Image credits: Meenakshi Sundaram K.

**Figure 4:**
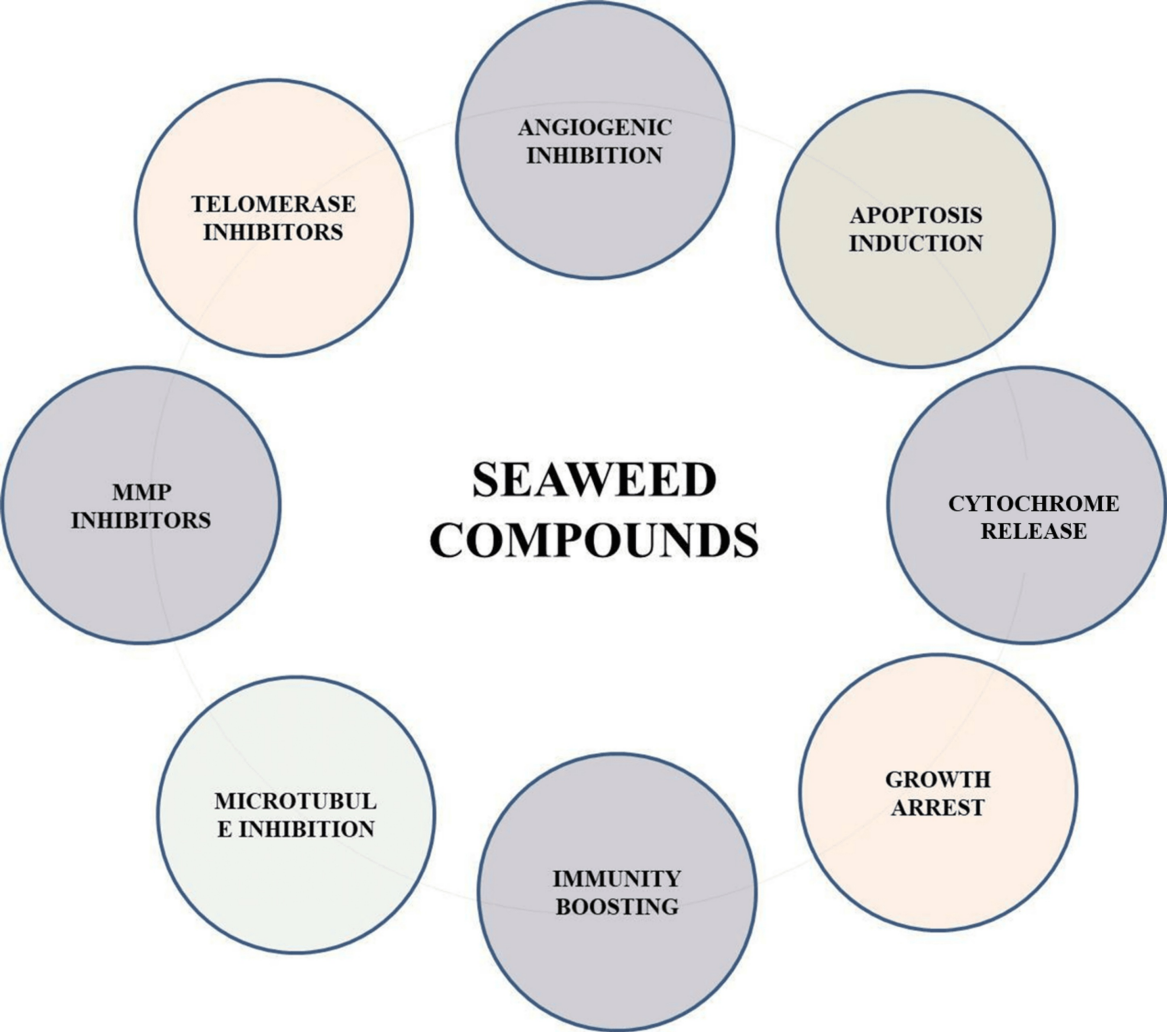
Seaweed compounds exert different modes of anti-cancer mechanisms. MMP: matrix metalloproteinases Image credits: Meenakshi Sundaram K.

Seaweeds continuously investigate environmental changes and factors such as radiation, salinity, and water levels throughout the day. These act as a driving force and contribute to the production of various compounds to adapt to their habitats. The composition and quantity of these bioactive compounds are determined by the species and geographical origin. Seaweeds are known to contain varying amounts of carbohydrates, proteins, phycobiliproteins, carotenoids, pigments, polyphenols, ash, lipids, terpenes, phlorotannins, and polysaccharides (Figure 3). Micronutrients such as zinc, iron, magnesium, manganese, and vitamins are also present in them. Eicosapentaenoic acid (EPA, 20:5n-3) and long-chain polyunsaturated essential fatty acids from the n-3 family (n-3 LC-PUFA) were found in many species to be present. These compounds are prominent drug resources against various physiological disorders, such as heart diseases, thrombosis, atherosclerosis, and carcinogenesis [2]. Recently, several authors reviewed (Figure 4) and emphasized the chemical nature of their anti-cancer activity (enzyme-treated seaweed extracts, glycolipids, glycoproteins, terpenes, steroids, and polyketide families for their inflammation, cell growth, cell-cell interaction, immune, and anticancer activities). The chemical composition was significantly different among the major classes of seaweeds. Brown algae have a high amount of fucosterol, which is the predominant sterol, while a low amount of cholesterol has been observed. In green algae, there is no single major sterol. According to the statement, brown seaweeds are superior to red ones in terms of their dietary and pharmacological uses. Various phenolic components and their derivatives, such as sargahydroquinoic acid, sargachromanol E, D, and polyunsaturated fatty acids such as palmitic acid, palmitoleic acid, elaidic acid, linoleic acid, linolenic acid, arachidonic acid, and cis-5, 8,11,14,17-eicosanoic acid, found in a variety of seaweeds, have been reported to play a significant role in anticancer activities [2-5] reported that sulfo lipids from seaweeds had antiproliferative activities against (human colon adenocarcinoma) DLD1 cells. The use of nanomaterials as drug delivery options has led to recent advancements in drug preparations and their subsequent isolation.

Extraction plays a major role in determining the bioactive nature of seaweed compounds

**Figure 5:**
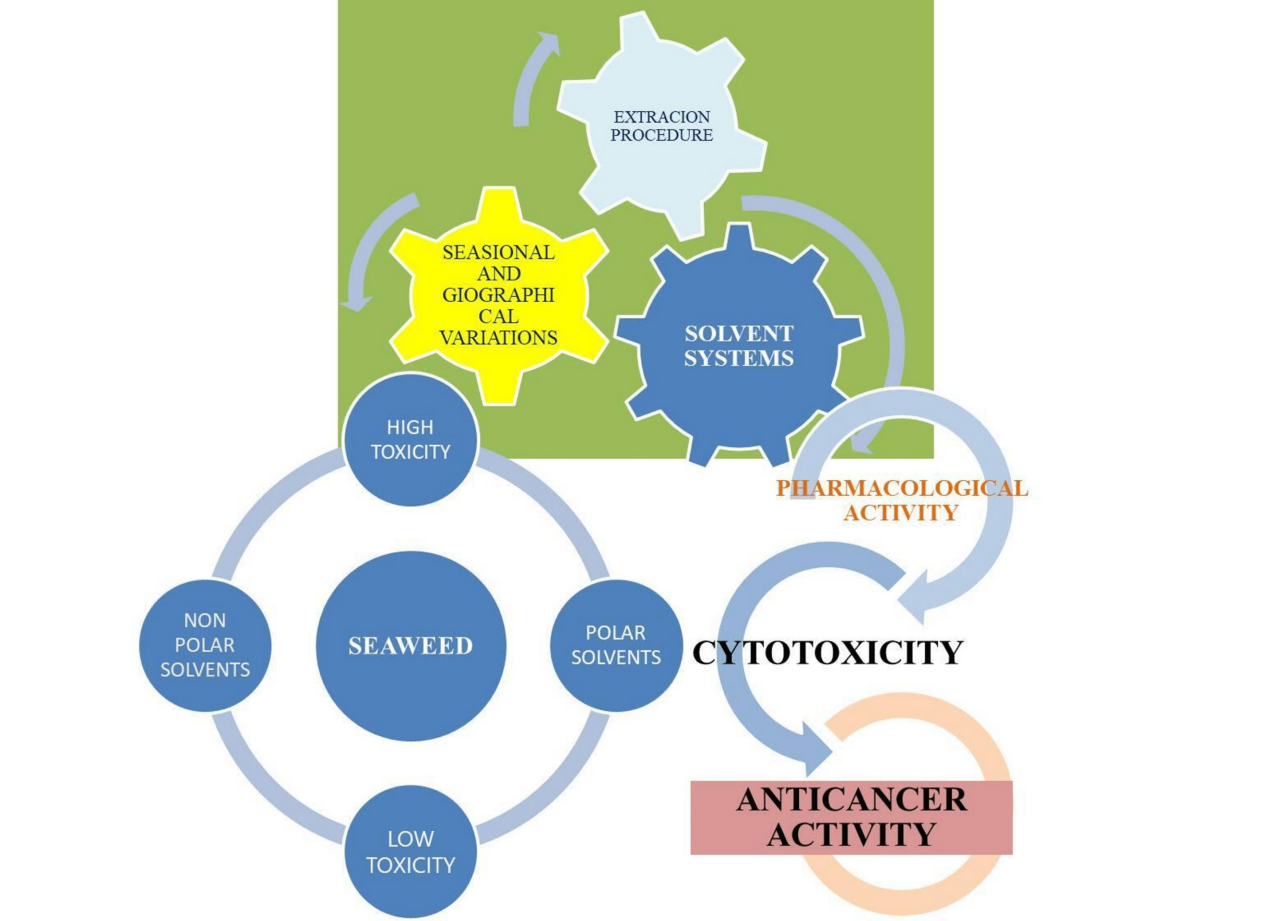
The extraction method strongly determines the nature of bioactivity in seaweed compounds. Seaweeds contain a variety of compounds, and the procedural variation is solely responsible for their pharmacological activities. Several studies showed that bioactive metabolites depend on the procedural variation of the extraction systems and are influenced by the geographical and seasonal parameters associated with the seaweeds. Image credits: Meenakshi Sundaram K.

The extraction procedure strongly determines the major proportion of the individual chemical compounds and is responsible for their bioactive nature (Figure 5). The solvents can change their pharmacological activity, and recent literature strongly emphasizes the selection of different solvent systems for seaweed extractions. Comparatively, the highest levels of cytotoxicity were associated with non-polar (hexane-based) algal extracts, while polar (methanol-based) extracts provide the lowest levels of cytotoxicity comparatively. The chloroform fraction of *Polysiphonia lanosa* showed nearly 10-fold more cytotoxicity than the methanolic extract [4]. Similarly, the non-polar hexane fraction was found to be more active than the polar solvent extraction of *Cystoseria myrica* against triple-negative breast cancer (MDAMB 486) cell lines [5]. Virtually hexane and chloroform extracts of *Laurencia catarinensis* and *Laurencia dendroidea* showed higher cytotoxicity than methanolic extracts. Thus, the extraction techniques play an important role in determining the bioactivity. The decades-long research on seaweed extractions brought novel developments and more insights into their properties. These developments lead to the usage of complex processes such as integration, intensification, and the development of sequential valorization of chemical chains as intact and preserve the nature of chemical entities to demand their natural bioactivity [4-5].

Microwave-assisted extraction (MAE), ultrasound-assisted extraction (UAE), supercritical fluid extraction (SFE), pressurized solvent extractions (PSE), reactive extrusion, and photo-bleaching [6] processes have reduced the involvement of solvents and provided the compounds with their actual chemical nature (maximally). Meanwhile, Mansur et al. (2020) [7] reported the seasonal variability in the anticancer activity of brown algae, *Cystoseira tamariscifolia* extract. They found significant variation in the bioactivity of the extract collected from the annual and/or different biotic cycles of the algae and showed that season should be an important factor for determining the bioactivity along with the extraction procedures. Thus, the bioactivity is mainly determined by the nature of extraction, isolated compounds, and related processes, and they need to be optimized depending on the particular algal species. The selection of the species should be determined with respect to its seasonal variation and different extraction procedures. The evaluation of traditional medicinal practices is one of the important clues for developing novel anti-cancer drug molecules, and in East Asian regions, a number of seaweeds have been utilized as important dietary materials for thousands of years. Recent studies showed the inverse association between algal intake (*Porphyra *sp.) and breast cancer risk in the East Asian women population. In recent decades, a broad series of compounds, including polysaccharides from edible seaweeds, have emerged as an important class of bioactive natural products, possessing many important properties of pharmacological relevance. The role of seaweed as a prebiotic catalyst for stimulating protective effects in phytoestrogen and estrogen metabolism is intriguing and provides a new opening that may be important in dietary chemoprevention of a broad range of cancers, especially breast cancer. Polysaccharides from seaweeds are involved in the activation of various cells, such as macrophages, neutrophils, T cells, and natural killer cells (NK cells), in view of immunotherapy. Dietary fucoxanthin is considered to be hydrolyzed to fucoxanthinol in the gastrointestinal tract by digestive enzymes, then absorbed into intestinal cells [2-4, 5-7]. 

Fucoxanthinol 1, (3’, 5'-dihydroxyphnoxy) -7- (2’’, 4’’, 6-trihydroxyphenoxy) -2,2,9-trihydroxydibenzo-1, 4-dioxin inhibited the expression of peroxisome proliferator-activated receptor gamma (PPaRγ), sterol regulatory element-binding transcription factor (SREBP1c), and CCAAT/enhancer binding proteins (C/EBP α) through an adipogenesis mechanism related to the downstream promoters of adipocyte-specific genes, including fatty acid-binding protein 4 (FABP4), fatty acid transport protein (FATP1), cell surface death receptor (FAS), lipoprotein lipase (LPL), acetyl CoA synthetase (ACS1), and leptin also reported likewise activity in colorectal cancer cells [8]. *Laurencia tristicha* extract had been reported to have anti-alkylation damage in peripheral lymphocytes due to antagonistic activity against DNA methylation. Ferreira et al. [9] reviewed the role of seaweed compounds on glioblastoma cells based on their anti-alkylating properties. Polyphenols of brown algal diet and topical application of extracts decreased ultraviolet B (UV-B)-induced skin tumor development with suppression of cyclooxygenase-2 (COX-2) expression with morbidity [9]. The average intake of edible algae in Japan was estimated to be 5.5 g/day. Based on this value, the average intake of fucoxanthin is calculated to be about 0.6 µmol/day. Beta-carotene, found in algal diets, can serve as an antioxidant or prooxidant, depending on its intrinsic properties as well as on the redox potential of the biological environment in which it acts. In animal and human studies, iodine administration has been shown to cause regression of both iodine-deficient goiter and benign pathological breast tissue [10]. Various types of brown algae, such as hijiki (*Sargassum fusiforme*), kombu (*Laminaria japonica*), and wakame (*Undaria pinnatifida*), are staples in the diet of East Asians. One of the main dietary differences between Japanese and Western women is the consumption of large amounts of iodine-rich seaweeds by the former, yielding a dietary iodine intake of several milligrams per day, which in turn reduces the risk of carcinogenesis [5-8].

## Review

Mechanism of action of seaweed-derived compounds towards anti-cancer activity

Apoptosis-Inducing Activity

**Figure 6:**
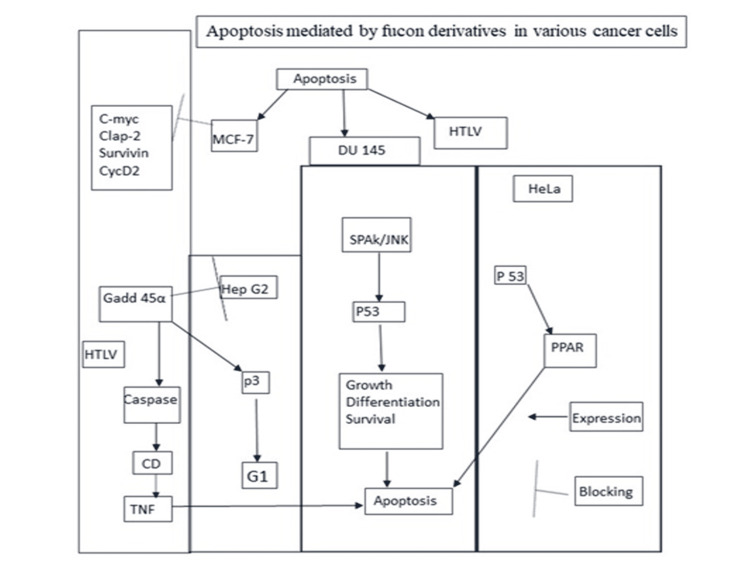
Fucon and their derivatives are one of the major kinds of polysaccharides in seaweed. These fucons target a variety of cell cycle checkpoints and cell signaling proteins that are mostly misregulated in many types of cancers. Particularly, they induce apoptosis in cancer cells by integrating different pathways in individual cancer cells. c-Myc: cellular myc; Clap 2: CARD-like apoptotic proteins; CARD: caspase-recruitment domains; Cyc D2: cyclin D2; MCF-7: Michigan Cancer Foundation-7; DU 145 cells: human prostate cancer cells; HTLV: human T-cell leukemia virus; TNF: tumor necrosis factor; CD: clusters of differentiation; GADD45α: growth arrest and DNA damage-inducible alpha; Hep G2: hepatocellular carcinoma; G1: gap phase; p3: tumor protein 3; p53: tumor protein 53; JNK: jun N-terminal kinase; HeLa: cervical cancer; PPAR: peroxisome-proliferator-activated receptors Image credits: Meenakshi Sundaram K.

As many of the cancer types improperly regulate or suppress the host apoptosis activity, apoptosis induction in the cancer microenvironment is a prime target for anti-cancer therapies. Recently, immense efforts have been made to reveal the novel functional roles of carotenoids, polyphenols, terpenes, glycolipids, and steroids from seaweeds and to identify their effective use in human health and disease prevention. They had shown several key mechanisms, including antioxidants, cell cycle arrest, induction of cell death, inhibition of metastasis, and angiogenesis, that were responsible for their anticancer activity (Figure 6). These compounds have wide structural diversity and have been shown to have complex biological potentials for regulating the important molecules that function in cancer processes. *Sargassum micracanthum* was shown to inhibit pro-inflammatory mediators via blockage of the nuclear factor kappa B (NF-β) signaling pathway. Polysaccharides from *Monostroma nitidum* were reported to increase the production of nitric oxide (NO) and prostaglandin E2 (PGE2) by up-regulating induced nitric oxide synthase (iNOS) and COX-2 in the murine macrophage cells [11]. Fucoidan was also shown to induce the expression of cluster of differentiation 40 (CD40), CD80, and CD86 for producing the interleukin-6 (IL-6), IL-12, and tumor necrosis factor-alpha (TNF-α) in spleen conventional dendritic cells (CDCs) in mice challenged with murine tumor cell in skin (B16-OVA) tumor cells. It also enhanced the interferon-gamma (IFN-γ) production and type 1 T helper (Th1) and tumor cell line (Tc1) cells in an IL-12-dependent manner, and that led to the increase in CD4 and CD8 T cells. Finally, ovalbumin (OVA) immunization with fucoidan as adjuvant protected mice from the challenge. Thus, Jin et al. had shown that fucoidan could be useful in cancer treatments and cancer vaccine development [12-14]. The anticancer natures of fucoidans have been reviewed by several authors, who recently demonstrated that the protease extract of *Ecklonia cava* activates the immune system via activation of the NF-κB pathway and IL-2 secretion for lymphocyte production [15]. *Porphyran *sp. extract was reported to regulate negatively insulin-like growth factor (IGF-IR) phosphorylation by decreasing the expression level of human gastric adenocarcinoma cell- lines (AGS gastric cancer cells) while inducing caspase-3 activation. the processes by which marine floral chemicals found that these mechanisms primarily depend on the activation of macrophages, the induction of apoptosis, and the prevention of oxidative damage to DNA due to the activation of numerous pathways [16].

The apoptosis-inducing effect of seaweed extracts seemed to relate strongly to the structural properties (Figure 2). Identifying the mode of cell death is recognized as a novel tactic for screening anticancer drugs. Active sulfated homo-heterofucans of brown seaweed have shown effective antitumor activities with a wide range of mechanisms. *Sargassum fulvellum* extract was found to mediate the up-regulation of a pro-apoptotic Bax protein and down-regulation of an anti-apoptotic B-cell leukemia/lymphoma 2 protein (Bcl-2 protein) and not by the immune system. Siphonaxanthin (and related compounds, such as siphonaxanthin, neoxanthin, violaxanthin, fucoxanthin, and astaxanthin), a specific keto-carotenoid from green algae, was reported to induce apoptosis in human leukemia (HL-60) cells and mature myeloid cells with up-regulation of p21 expression mediated by and as beta-carotene [17-18]. Sulfate content of fucoidan had been shown to be a stronger inducer of apoptosis associated with degradation poly(ADP-ribose) polymerase (PARP) and caspase-3 and -7 activation in (human myeloid leukemia cell line) U937 cells [17-19]. Similarly, several authors showed the existence of a strong relationship between the anticancer mechanisms and the chemical structures [19]. 

The structural diversity of quercetin (Que) had a significant range of effects in Michigan Cancer Foundation-7 (MCF-7) cells significantly and was able to exhibit different growth arrest patterns based on the duration [14-18]. Costa et al. (2011) reported that heterofucan (fraction) steroidogenic factor 1 (SF-1.5v) from *Sargassum filipendula* induced the release of the apoptosis-inducing factor (AIF) from mitochondria and enhanced the apoptosis in HeLa cells [20]. Similar activity was also shown by edible seaweed, *Halopteris scoparia*, and *Sargassum coreanum* [5-10]. Peroxisome proliferator-activated receptor-gamma (PPAR-c) is a ligand-activated transcription factor that has been implicated in many processes related to cellular development, differentiation, and physiology and is expressed in many human cancers, including breast, colon, stomach, prostate, pancreas, bladder, placenta, lung, chondrosarcoma, and leukemia. Hesperidin, a flavanone glycoside usually found in the citrus family, was also reported in Moroccan seaweeds. It was shown to regulate lipid metabolism and glucose metabolism by mediating AMP-activated protein kinase (AMPK) and peroxisome proliferator-activated receptors (PPAR) signaling pathways, and as a result, it was able to modulate the antioxidant index and anti-apoptosis pathways oriented towards NF-κB signaling to play a significant role in inflammations [18-21]. Chromene, a metroditerpinoid from *Sargassum *sp., was found to upregulate the Bax, activate caspase 3, and downregulate Bcl-xL with the cleavage of PARP in HL-60 cells. Further, Agrody et al. (2020) showed that chromene derivatives exhibited strong anticancer activity in a number of cancer cell lines, such as the mammary gland breast cancer cell line (MCF-7), human colon cancer (HCT-116), and liver cancer (HepG-2) by arresting the growth at the G2/M phases and triggering apoptosis [22]. PARP is one of the apoptosis-induced mechanisms reported in the seaweed-treated cancer cell lines. Besides their extract also regulating the cyclin-dependent kinase inhibitor 1A (p21WAF1/CIP1), Jun-Proto-Oncogene (c-Jun), and Jun N-terminal kinase (JNK) pathways in breast cancer (MCF cells) [18-23]. 

Activation of Antiapoptotic Protein

The tumor microenvironment is characterized by key physiologically disturbing conditions such as hypoxia and low nutrients and leads to the accumulation of unfolded proteins that eventually result in endoplasmic reticulum (ER), so-called ER stress. Thus, modulating the ER stress pathway is one of the key anti-cancer strategies. Most of the natural compounds are shown to have anti-cancer effects via ER stress. Interestingly, there are vast studies that address the different natures of such compounds with different cell lines, viz. lung, breast, colorectal, gastric, prostate, and liver cancer. Sharma et al. showed that the phenolic extract of *Halophila ovalis *decreased the anti-apoptotic proteins such as Bcl-2 and Nrf-2 in breast cancer (MCF-7) cells [24]. The AIF is a flavin adenine dinucleotide (FAD)-containing, nitinamide-adenine dinucleotide hydrogen (NADH)-dependent oxidoreductase found in the mitochondrial intermembrane space. It induces phosphatidylserine exposure on the cell surface. It might also maintain apoptogenic ability in the presence of the pancaspase inhibitor. Several key anti-cancer mechanisms of sulfated polysaccharides, such as human papillomavirus (HPV) inhibition, were reviewed. λ-, cyclized μ/ι-carrageenans have been shown to inhibit virus attachment, viral internalization, uncoating, transcription, and replication as a main anti-cancer mechanism against HPV. Heterofucans and terpenoids from brown algae are shown to be promising agents with anti-cervical cancer activity. Thermostable heterofucan steroidogenic factor (SF)-1.5v from *Sargassum filipendula*-induced apoptosis, via mitochondrial release of AIF into cytosol, and caspase-dependent manner by decreasing Bcl-2 and increasing the bax with glycogen synthase kinase (GSK) dephospharylation, in HeLa cells [14-20]. 

Inhibition of Angiogenesis

**Figure 7:**
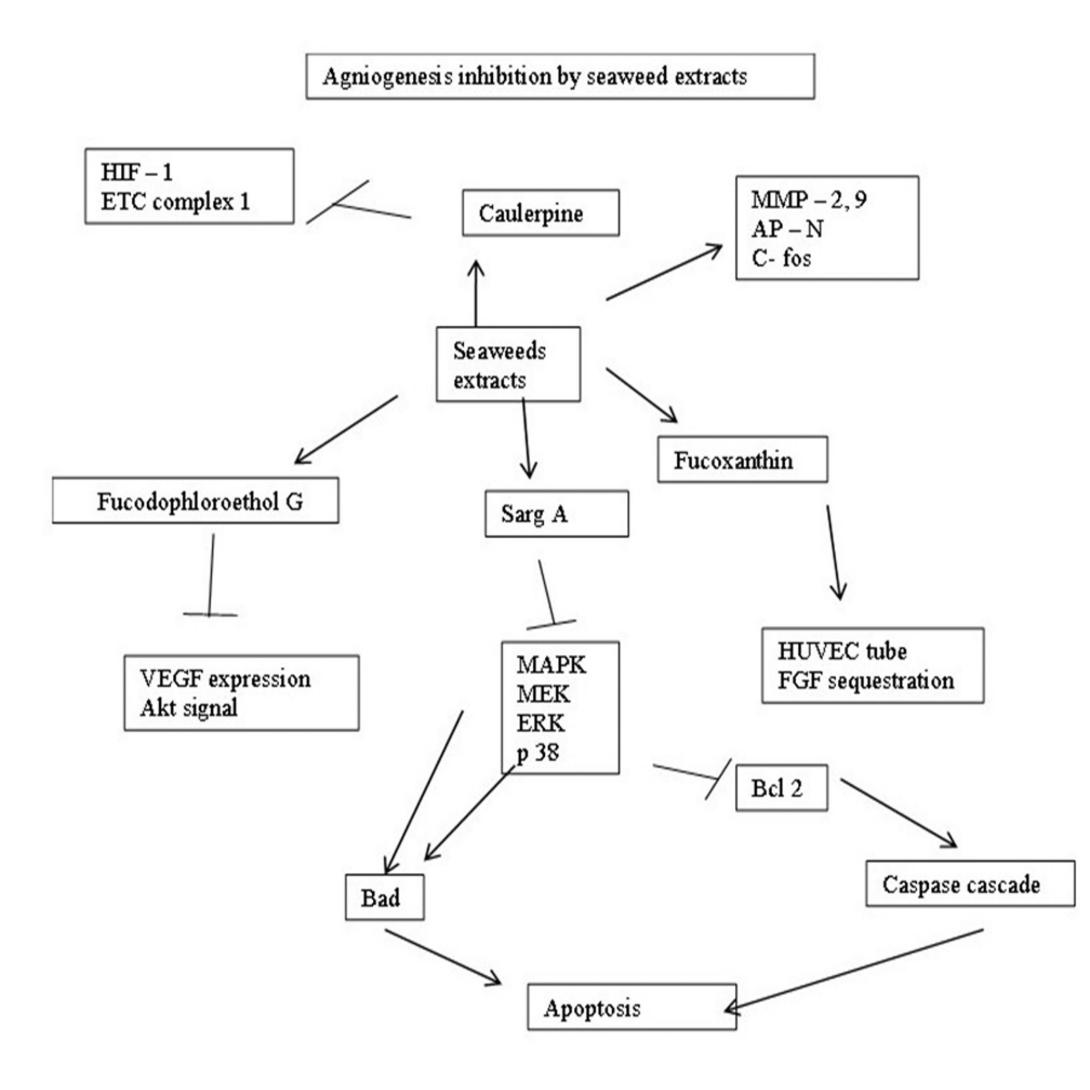
Angiogenesis inhibition by seaweed extracts and their components HIF: hypoxia-inducible transcription factors; ETC: effusion tumor cell; MMP-2: matrix metalloproteinases; APN: aminopeptidase N; C-fos: proto-oncogene c-fos; SARG: specifically androgen-regulated gene; VEGF: vascular endothelial growth factor; MAPK: mitogen-activated protein kinase; MEK: mitogen-activated extracellular signal-regulated kinase; ERK: extracellular signal-regulated kinases; p38: protein kinase 38; HUVEC: human umbilical vein endothelial cell; FGF: fibroblast growth factors; BAD: BCl2-associated agonist of cell death; BCl2: B-cell leukemia/lymphoma 2 protein Image credits: Meenakshi Sundaram K.

Deregulated angiogenesis is the main cause of cancer growth, and anti-angiogenic therapy is a promising strategy for anti-cancer activity. Currently, available Food and Drug Administration (FDA)-approved drugs are targeting either vascular endothelial growth factor or its receptor, but in the long term, these approaches were shown to cause several side effects, and the chances of developing resistance to these drugs are also high. Therefore, the identification of safe and cost-effective anti-angiogenic molecules is highly imperative. Over the past decades, dietary-based natural compounds have been studied for their anti-angiogenic potential, which provided avenues for improving angiogenesis-based therapy [6-8]. Several antioxidants found in seaweeds have been reported to inhibit tumor angiogenesis, viz., N-acetyl cysteine (NAC) inhibits tumor angiogenesis via suppressing the production of vascular endothelial growth factor (VEGF) and by promoting angiostatin, a potent endothelial apoptotic factor [10]. The epithelial-to-mesenchymal transition (EMT) is a mechanism by which solid tumors become metastatic [25]. An ideal antiangiogenic inhibitor should be able to induce apoptosis in the EMT transition, so an ideal anti-angiogenic drug should be anti-tumor epithelium in nature. Many seaweed metabolites enhance the expression of native angiogenic inhibitors such as angiostatin, endostatin, interferons, IL-1, and IL-12, tissue inhibitors of metalloproteinases, retinoic acid, etc. However, the tumors are self-sufficient in vessel formation, migration, and apoptosis inhibition due to the chemoresistant [24-26]. So such inhibitors should be able to inhibit tumor vascularization in such a kind of altered cell physiological condition. The ratio of pro-angiogenesis and anti-angiogenesis regimes determines the vascular homeostasis [27]. The proangiogenic approaches lead to pericyte stabilization and warrant a mature vascular network for effective drug transportation [28-30]. Meanwhile, the cytotoxic drugs may induce tumor toxicity by the apoptotic pathways. Such drug candidates should have a pro-apoptotic nature in stabilizing the blood vessels, and at the same time, they should have antitumor activity [24-26]. The antiangiogenic nature of seaweed compounds has been summarized in Figure 7. 

## Conclusions

Novel anti-cancer therapies can be developed with the help of seaweeds and their secondary metabolites. The diverse bioactive compounds in seaweeds exhibit anti-cancer properties through multiple molecular mechanisms. The inhibition of cancer cell proliferation, apoptosis, and tumor angiogenesis by these compounds has shown great potential for cancer treatment. Primary compounds like fucoidans, phlorotannins, and polysaccharides have been proven to be effective in addressing diverse molecular pathways implicated in cancer progression. Modulation of immune responses and suppression of the phosphatidylinositol 3-kinase (PI3K/Akt) and mitogen-activated protein kinase (MAPK) signaling pathways, which are essential for cell survival and proliferation, is what Fucoidans do to achieve their anti-cancer properties. Seaweed polysaccharides interfere with the Wingless-related integration site (Wnt)/Uterine natural killer cells<(unk)> -catenin signaling pathway, resulting in a decrease in cancer cell viability and tumor growth. Inhibition of the Wnt/<unk>-catenin signaling pathway by seaweed polysaccharides has been demonstrated, leading to a reduction in cancer cell viability and tumor growth. Seaweed-derived compounds can be used as multi-target agents in cancer therapy, as demonstrated by these molecular mechanisms. Due to their uniqueness and ability to disrupt critical pathways involved in tumor development and metastasis, they are a compelling alternative or complement to traditional chemotherapeutic agents. Future research should focus on further elucidating the detailed molecular interactions of these compounds, optimizing their extraction and purification processes, and conducting comprehensive clinical trials to validate their efficacy and safety in humans. Improving our understanding of seaweed metabolites and their mechanisms of action can lead to the discovery of new avenues for effective cancer therapy and improved therapeutic outcomes for patients. In short, seaweeds and their secondary metabolites are highly promising as novel cancer-fighting agents, to put it succinctly. The multiple mechanisms of action they possess against cancer and tumor angiogenesis can make it easier to develop innovative therapies that could enhance the current landscape of cancer treatment.

## References

[1] Suleria HA, Osborne S, Masci P, Gobe G (2015). Marine-based nutraceuticals: an innovative trend in the food and supplement industries. Mar Drugs.

[2] Karthik G (2021). Knowledge and awareness on the association between physical inactivity, junk food consumption and obesity among adolescent population- a survey based analysis. Int J Dentistry Oral Sci.

[3] Erika M, Danil X, Cleidiane S (2011). Search for Cytotoxic agents in multiple Laurencia complex Seaweed species (Ceramiales, Rhodophyta) harvested from the Atlantic Ocean with emphasis on the Brazilian state of Espírito Santo. Rev Bras Farmacogn.

[4] Leandro A, Pacheco D, Cotas J, Marques JC, Pereira L, Gonçalves AM (2020). Seaweed’s bioactive candidate compounds to food industry and global food security. Life (Basel).

[5] Eom SJ, Kim YE, Kim JE (2020). Production of Undaria pinnatifida sporophyll extract using pilot-scale ultrasound-assisted extraction: extract characteristics and antioxidant and anti-inflammatory activities. Algal Res..

[6] Shoeib NA, Bibby MC, Blunden G, Linley PA, Swaine DJ, Wheelhouse RT, Wright CW (2004). In-vitro cytotoxic activities of the major bromophenols of the red alga Polysiphonia lanosa and some novel synthetic isomers. J Nat Prod.

[7] Mansur AA, Brown MT, Billington RA (2010). The cytotoxic activity of extracts of the brown alga Cystoseira tamariscifolia (Hudson) Papenfuss, against cancer cell lines changes seasonally. J Appl Phycol.

[8] Ishikawa C, Tafuku S, Kadekaru T (2008). Anti-adult T-cell leukemia effects of brown algae fucoxanthin and its deacetylated product, fucoxanthinol. Int J Cancer.

[9] Ferreira J, Ramos AA, Almeida T, Azqueta A, Rocha E (2018). Drug resistance in glioblastoma and cytotoxicity of seaweed compounds, alone and in combination with anticancer drugs: a mini review. Phytomedicine.

[10] Kavalappa YP, Rudresh DU, Gopal SS (2019). β-carotene isolated from the marine red alga, Gracillaria sp. potently attenuates the growth of human hepatocellular carcinoma (HepG2) cells by modulating multiple molecular pathways. J Funct Foods.

[11] Karnjanapratum S, You S (2011). Molecular characteristics of sulfated polysaccharides from Monostroma nitidum and their in vitro anticancer and immunomodulatory activities. Int J Biol Macromol.

[12] Jin JO, Zhang W, Du JY, Wong KW, Oda T, Yu Q (2014). Fucoidan can function as an adjuvant in vivo to enhance dendritic cell maturation and function and promote antigen-specific T cell immune responses. PLoS One.

[13] Jin JO, Chauhan PS, Arukha AP, Chavda V, Dubey A, Yadav D (2021). The therapeutic potential of the anticancer activity of fucoidan: current advances and hurdles. Mar Drugs.

[14] Teruya T, Konishi T, Uechi S, Tamaki H, Tako M (2007). Anti-proliferative activity of oversulfated fucoidan from commercially cultured Cladosiphon okamuranus TOKIDA in U937 cells. Int J Biol Macromol.

[15] Kuznetsova TA, Persiyanova EV, Ermakova SP, Khotimchenko MYu, Besednova NN (2018). The sulfated polysaccharides of brown algae and products of their enzymatic transformation as potential vaccine adjuvants. Nat. Prod. Commun.

[16] Dai YL, Jiang YF, Lee HG, Jeon YJ, Kang MC (2019). Characterization and screening of anti-tumor activity of fucoidan from acid-processed hijiki (Hizikia fusiforme). Int J Biol Macromol.

[17] (2019). Bioactive Natural Products for the Management of Cancer: From Bench to Bedside. Bioactive Natural Products for the Management of Cancer: from Bench to Bedside.

[18] Singh K (2019). Immunomodulatory and therapeutic potential of marine flora products in the treatment of cancer. Bioactive Natural Products for the Management of Cancer: From Bench to Bedside.

[19] Wang Y, Xing M, Cao Q, Ji A, Liang H, Song S (2019). Biological activities of fucoidan and the factors mediating its therapeutic effects: a review of recent studies. Mar Drugs.

[20] Silva Costa L, Silva Telles CB, Medeiros Oliveira R (2011). Heterofucan from Sargassum filipendula induces apoptosis in HeLa cells. Mar Drugs.

[21] Hussain A, Bourguet-Kondracki ML, Majeed M (2023). Marine life as a source for breast cancer treatment: a comprehensive review. Biomed Pharmacother.

[22] El-Agrody AM, Fouda AM, Assiri MA (2020). In vitro anticancer activity of pyrano [3, 2-c] chromene derivatives with both cell cycle arrest and apoptosis induction. Med Chem Res.

[23] Del Mondo A, Smerilli A, Ambrosino L, Albini A, Noonan DM, Sansone C, Brunet C (2021). Insights into phenolic compounds from microalgae: structural variety and complex beneficial activities from health to nutraceutics. Crit Rev Biotechnol.

[24] Sharma H, Stephen NM, Gopal SS (2021). Phenolic extract of seagrass, Halophila ovalis activates intrinsic pathway of apoptosis in human breast cancer (MCF-7) cells. Nutr Cancer.

[25] Lee JH, Park SE, Hossain MA (2007). 2,3,6-tribromo-4,5-dihydroxybenzyl methyl ether induces growth inhibition and apoptosis in MCF-7 human breast cancer cells. Arch Pharm Res.

[26] Ratchanee K, Raymond J, Anchana P (2016). Variations of tidal exposures and seasons on growth, morphology, anatomy and physiology of the seagrass Halophila ovalis (R.Br.) Hook. f. in a seagrass bed in Trang Province, Southern Thailand. Aquat Bot.

[27] Seong SH, Paudel P, Jung HA, Choi JS (2019). Identifying phlorofucofuroeckol-A as a dual inhibitor of amyloid-β(25-35) self-aggregation and insulin glycation: elucidation of the molecular mechanism of action. Mar Drugs.

[28] Aisa Y, Miyakawa Y, Nakazato T, Shibata H, Saito K, Ikeda Y, Kizaki M (2005). Fucoidan induces apoptosis of human HS-sultan cells accompanied by activation of caspase-3 and down-regulation of ERK pathways. Am J Hematol.

[29] Lospinoso Severini L, Ghirga F, Bufalieri F, Quaglio D, Infante P, Di Marcotullio L (2020). The SHH/GLI signaling pathway: a therapeutic target for medulloblastoma. Expert Opin Ther Targets.

[30] Li YX, Li Y, Qian ZJm, Ryu B, Kim SK (2011). Suppression of vascular endothelial growth factor (VEGF) induced angiogenic responses by fucodiphloroethol G. Process Biochem.

